# Retraction: Baicalin induced dendritic cell apoptosis *in vitro*

**DOI:** 10.3389/fphar.2014.00283

**Published:** 2014-12-17

**Authors:** Frontiers in Pharmacology Editorial Office

The journal is retracting the 29 March 2011 article cited above. Based on information reported after publication, this article was found to contain incorrect data and invalid statistical analyses.

The journal and chief editor have therefore decided to retract the article in its entirety and apologize to the readers of Frontiers in Pharmacology.

The corresponding author agrees with the retraction of the article and also apologizes to the readers.

